# Hypoxia, but not an electrolyte-imbalanced diet, reduces feed intake, growth and oxygen consumption in rainbow trout (*Oncorhynchus mykiss*)

**DOI:** 10.1038/s41598-018-23352-z

**Published:** 2018-03-21

**Authors:** Leonardo J. Magnoni, Ep Eding, Isabelle Leguen, Patrick Prunet, Inge Geurden, Rodrigo O. A. Ozório, Johan W. Schrama

**Affiliations:** 1 CIIMAR, University of Porto, Matosinhos, 4450-208 Portugal; 2 IIB-INTECH, CONICET-UNSAM, Chascomús, 7310 Argentina; 3 AFI, WIAS, Wageningen University, Wageningen, 6700 AH The Netherlands; 4 LPGP-INRA, Université Rennes, Rennes, 35042 France; 5 NuMeA-INRA, St Pée-sur-Nivelle, 64310 France; 6 ICBAS, University of Porto, Porto, 4050-313 Portugal

## Abstract

Oxygen limitation and dietary imbalances are key aspects influencing feed intake (FI) and growth performance in cultured fish. This study investigated the combined effects of hypoxia and dietary electrolyte balance on the growth performance, body composition and nutrient utilization in a rainbow trout (*Oncorhynchus mykiss*) isogenic line. Fish were fed *ad libitum* two experimental diets: electrolyte-balanced or -imbalanced diets (DEB 200 or 700 mEq kg^−1^, respectively) and exposed to normoxia or hypoxia (7.9 or 4.5 mg O_2_ l^−1^, respectively) for 42 days. DEB did not affect FI, growth performance or body composition. Nevertheless, hypoxia had a negative impact, reducing FI (6%), growth rate (8%), oxygen consumption (19%), energy (5%) and lipid (42%) contents. Digestible energy intake and heat production were higher in normoxic fish (40% and 23%, respectively), retaining 64% more energy in lipid or protein. Hypoxia reduced the apparent digestibility of dry matter, ash, protein, lipid, carbohydrates and energy. Trout fed DEB 700 diet were energetically less efficient, reflected in higher heat production and energy requirements for maintenance. FI was inhibited by low dissolved oxygen levels, but not by electrolyte-imbalanced diet, in spite of the higher energy requirements for maintenance. This study highlights the importance that dietary-electrolyte content and DO levels have on energy balance and growth performance when fish are fed to satiation.

## Introduction

Feed intake (FI) is the main determinant of animal growth. In fish, voluntary FI is influenced by dietary, environmental and/or physiological factors. The impact of dissolved oxygen (DO) level on FI has been widely documented in different fish species^1–4^, including the rainbow trout^5,6^. In general, FI decreases with decreasing DO, as this physiological trait is limited by the oxygen uptake capacity^7^. At normoxia and in the absence of other constraints, the long term (weeks) FI of fish can be constrained by a set-point value of oxygen consumption. It is proposed that diet composition may affect the amount of oxygen consumed per unit of feed and this may posse further limits in fish subjected to hypoxia^8^. However, marked intraspecific variation in the metabolic phenotype of fishes has been described, which are suggested to be influenced by both genes and developmental conditions^9^.

Fish may display metabolic depression as part of an adaptive response to a stress situation^10^ (e.g. hypoxia), resulting in downregulation of metabolic activity by multiple signalling factors at tissue and cellular levels^11^. European sea bass (*Dicentrarchus labrax*) chronically exposed to low DO and crowding have been shown to decrease their FI and energy requirements for maintenance^12^. Similarly, energy requirements for maintenance in Nile tilapia (*Oreochromis niloticus*) were reduced when exposed to low DO levels^4^. In particular, it has been suggested that energy requirements for maintenance could be lessened in rainbow trout (*Oncorhynchus mykiss*) exposed to hypoxia^5^, implying that nutrient utilization could be modulated by DO levels. Thus, fish exposed to sub-optimal conditions, including low DO, reduce their FI and partially compensate this with more efficient nutrient utilization. Nevertheless, the combined effects of hypoxia and diet composition on dietary nutrient utilization are still poorly understood.

Saravanan *et al*.^13^ showed that both hypoxia (DO 4.0 mg O_2_ l^−1^) and an amino-acid-imbalance diet produced a significant reduction in FI in rainbow trout. However, the study showed that oxygen consumption in trout were unaffected by the dietary imbalance. Similarly, the oxygen consumption in Nile tilapia were not altered by dietary lipid to carbohydrate ratios, while FI was affected^14^. Both studies suggest that nutritional factors affecting energy use and oxygen availability could pose constraints on FI, although fish may have effective mechanisms to compensate for changes in energy intake in order to achieve energy balance.

The acid-base homeostasis (pH) is one of the most important physiological processes in fish, while dietary and environmental conditions may deeply alter this balance^15^. Acid-base homeostasis disturbances appear to increase the oxygen consumption required for maintenance metabolism in fish, as the animal needs several energy consuming processes essential to keeping this balance. In fact, the oxygen consumption rate increases in rainbow trout reared at pH either below or above the optimal pH^16^. Regulation of systemic pH is then achieved by adjusting the rates of acid and/or base excretion, which in turn are linked to ion uptake through the involvement of ions exchange mechanism occurring mainly in the gill^17–19^. The kidney plays a complementary role in acid-base balance by reabsorbing HCO_3_^−^ from the filtrate^18^. In addition, the enzyme carbonic anhydrase present in gill and kidney is involved in the regulation of acid-base balance of freshwater rainbow trout^20^.

In fish, the acid-base homeostasis can be affected by the dietary electrolyte balance (DEB) which in turn will alter the maintenance energy expenditure^21,22^. The DEB is defined as the sum of the mineral cations minus the sum of mineral anions present in the diet. Differences in DEB may occur when feed ingredients containing different quantities of cations (Na, K, Ca and Mg) and anions (Cl and P) are included in the diet formulation^23^. A low DEB diet has acidic properties, while a high DEB diet has alkaline properties. Alterations in DEB can trigger mechanisms to counteract acid-base imbalances. The gastro-intestinal tract (GIT) and gills produce acid-base secretions for the reestablishment of the acid-base homeostasis, at the expense of an extra energetic cost. Previous studies in Nile tilapia and meagre fed either 200 DEB or electrolyte- imbalanced diets (800 and 700 DEB, respectively) have shown that maintenance energy expenditure increases with DEB level^24,25^. However, studies on the impact of DEB on growth, FI and nutrient digestibility in fish have been limited, in spite of the overlapping roles of the GIT in water/ionic balance and nutrient digestion/assimilation. Feeding fish with different DEB may enable us to elucidate the limits of the oxygen consumption, energy use and availability which are required for growth, having important practical implications for fish production.

The current study investigated the changes in FI on growth performance, oxygen consumption, nutrient digestibility, nitrogen and energy balance, together with several metabolic markers in an isogenic heterozygous family of rainbow trout (*Oncorhynchus mykiss*) subjected to a combination of nutritional (DEB 200 or DEB 700) and environmental (normoxia or hypoxia) challenges. An isogenic trout line was employed in this study for their genetic uniformity, providing an experimental model with low intra-specific variability, thus high reproducibility.

## Results

### Feed intake, growth performance and oxygen consumption

**Figure 1:**
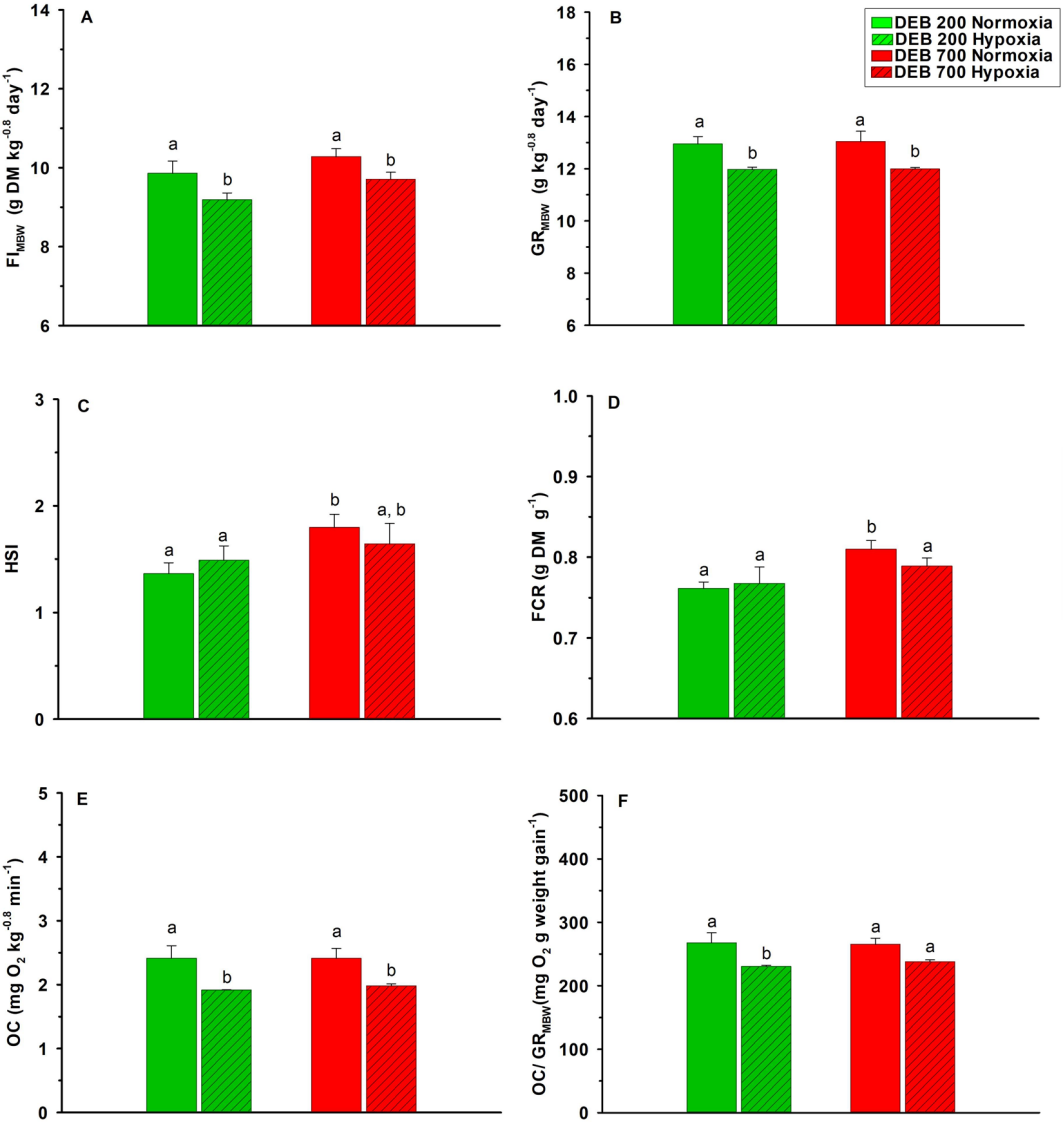
Effects of dietary electrolyte balance (DEB) and dissolved oxygen levels on (**A**) feed intake expressed per unit of metabolic body weight (FI_MBW_); (**B**) growth rate expressed in metabolic body weight (GR_MBW_); (**C**) hepatosomatic index (HSI); (**D**) feed conversion ratio (FCR); (**E**) oxygen consumption (OC) and (**F**) oxygen consumption to growth rate (OC/GR_MBW_) of rainbow trout. Further details are provided in Methods. Values are mean ± SEM (n = 3 tanks, except for HSI where n = 9 fish). Different letters indicate differences among treatments (α = 0.050).

Figure 1 presents FI expressed per unit of metabolic body weight (FI_MBW_), growth rate expressed by metabolic body weight (GR_MBW_), hepato-somatic index (HSI), feed conversion ratio (FCR), oxygen consumption (OC) and the coefficient between oxygen consumption and growth rate (OC/GR_MBW_). FI_MBW_, GR_MBW_, OC or OC/GR_MBW_ were not affected by the diet, but were altered by DO levels (P < 0.01) (Table 1). FI_MBW_ (6.7%) and GR_MBW_ (1.2%) were higher in normoxic when compared to hypoxic groups. Also, OC and the relation between OC and GR_MBW_ were significantly higher in normoxia than in hypoxia (24% and 14%, respectively). On the other hand, HIS and FCR were not altered by DO level, but were 16% and 4.6% respectively higher in fish fed the DEB 700 diet (P < 0.05). No interactions between DEB and DO levels were observed for these parameters. No mortalities were recorded during the trial, except in the DEB 200 treatment in hypoxic condition (98.9% survival).

**Table 1:** Two-way ANOVA analysis on the effects of diet (DEB) and dissolved oxygen levels (DO) and their respective interactions on growth performance and body indexes in trout. Feed intake is expressed per unit of metabolic body weight (FI_MBW_); growth rate is expressed in per unit of metabolic body weight (GR_MBW_); Hepato-somatic index (HSI); Feed conversion ratio (FCR); Oxygen consumption (OC); Coefficient between oxygen consumption and growth rate expressed on metabolic body weight (OC/GR_MBW_); Interaction (I); Not significant (ns) P > 0.1; *P < 0.05; **P < 0.01.

Parameters	Factors		
	DEB	DO	I
FI_MBW_	ns	*	ns
GR_MBW_	ns	**	ns
HSI	*	ns	ns
FCR	*	ns	ns
OC	ns	**	ns
OC/GR_MBW_	ns	**	ns

### Body composition

Dietary treatment did not affect body composition, while low DO levels decreased dry matter, lipids and energy (P < 0.05) (Table 2). Normoxic fish had 9% more lipids and about 4.5% more energy than hypoxic fish. The DEB and DO interaction did not significantly affect body nutrient (proteins, lipids) and energy content.

**Table 2:** Effects of dietary electrolyte balance (DEB) and dissolved oxygen levels (DO) on body weight (BW) and composition in trout. DEB in mEq kg^−1^. Parameters are expressed as g kg^−1^, except for BW and energy, which are expressed in g and kJ g^−1^, respectively (all as wet fish weight). Mean values per tank (n = 3), measured in 30 fish. Further details are provided in Methods. Pooled standard error of mean (SEM); Interaction (I); Not significant (ns) P > 0.1; *P < 0.05; **P < 0.01.

Parameters	DEB 200		DEB 700					
	Normoxia	Hypoxia	Normoxia	Hypoxia	SEM	DEB	DO	I
Initial BW	116	114	115	116	2.0	ns	ns	ns
Final BW	207	185	215	187	6.6	ns	**	ns
DM	299	288	296	286	3.2	ns	*	ns
Ash	21	21	21	22	0.4	ns	ns	*
Phosphorous	3.96	3.89	3.83	3.94	0.09	ns	ns	ns
Protein	174	174	175	174	2.2	ns	ns	ns
Lipid	104	97	102	92	3.0	ns	*	ns
Energy	8.2	7.8	7.9	7.6	0.15	ns	*	ns

### Nutrient digestibility, nitrogen and energy balances

The apparent nutrient and energy digestibility coefficients are presented in Table 3 and are used to calculate the nitrogen and energy balance parameters presented in Tables 4 and 5, respectively. DEB affected the digestibility of dry matter (P < 0.001), ash (P < 0.001), lipid (P < 0.001) and energy (P < 0.05), but did not affect protein and carbohydrate digestibility coefficients. DO level affected the digestibility of dry matter (P < 0.001), ash (P < 0.05), proteins (P < 0.001), lipids (<0.01), carbohydrates (P < 0.001) and energy (P < 0.01). However, no interaction between the DEB and DO levels was detected.

**Table 3:** Effects of dietary electrolyte balance (DEB) and dissolved oxygen levels (DO) on the apparent nutrient digestibility in trout. DEB in mEq kg^−1^. Parameters are expressed as %. Mean values per tank (n = 3), measured in 30 fish. Further details are provided in Methods. Pooled standard error of mean (SEM); Interaction (I); Not significant (ns) P > 0.1; *P < 0.05; **P < 0.01; ***P < 0.001.

Parameters	DEB 200		DEB 700					
	Normoxia	Hypoxia	Normoxia	Hypoxia	SEM	DEB	DO	I
DM	84.4	86.0	85.7	87.9	0.18	***	***	ns
Ash	32.2	33.0	52.0	54.9	0.66	***	*	ns
Phosphorous	48.6	47.7	41.9	42.9	0.85	***	ns	ns
Protein	96.4	97.2	96.4	97.3	0.09	ns	***	ns
Lipid	97.3	97.7	96.5	96.9	0.09	***	**	ns
Carbohydrates	73.4	77.1	72.7	77.7	0.35	ns	***	ns
Energy	91.5	92.9	90.9	92.7	0.12	*	***	ns

**Table 4:** Effects of dietary electrolyte balance (DEB) and dissolved oxygen levels (DO) on nitrogen balance in trout. DEB in mEq kg^−1^. Gross nitrogen intake (GN); digestible nitrogen (DN); branchial and urinary nitrogen excretion (BUN); retained nitrogen (RN) expressed in kJ kg^−0.8^ day^−1^. Protein efficiency (PE) expressed as %. Further details are provided in Methods. Mean values per tank (n = 3), measured in 30 fish. Pooled standard error of mean (SEM); Interaction (I); Not significant (ns) P > 0.1; *P < 0.05; **P < 0.01; ***P < 0.001.

Parameters	DEB 200		DEB 700					
	Normoxia	Hypoxia	Normoxia	Hypoxia	SEM	DEB	DO	I
GN	536	373	569	402	41.8	ns	**	ns
DN	517	362	548	391	40.3	ns	**	ns
BUN	218	147	233	175	13.2	ns	**	ns
RN	299	215	315	216	27.9	ns	*	ns
PE	57.6	59.4	57.2	55.3	1.2	*	ns	ns

**Table 5:** Effects of dietary electrolyte balance (DEB) and dissolved oxygen levels (DO) on energy balance in trout. DEB in mEq kg^−1^. Gross energy intake (GE); digestible energy (DE); branchial and urinary energy excretion (BUE); metabolizable energy (ME); heat production (HP); retained energy (RE) and metabolizable energy for maintenance (MEm) are expressed in kJ kg^−0.8^ day^−1^. Mean values per tank (n = 3), measured in 30 fish. Further details are provided in Methods. Pooled standard error of mean (SEM); Interaction (I); Not significant (ns) P > 0.1; *P < 0.05; **P < 0.01; ***P < 0.001.

Parameters	DEB 200		DEB 700					
	Normoxia	Hypoxia	Normoxia	Hypoxia	SEM	DEB	DO	I
GE	168	117	178	125	13.1	ns	**	ns
DE	153	108	161	116	11.9	ns	**	ns
BUE	5.4	3.6	5.8	4.3	0.33	ns	**	ns
ME	148	105	156	112	11.6	ns	**	ns
HP	51	39	61	52	3.0	**	**	ns
RE	97	65	95	60	9.2	ns	**	ns
MEm	19	18	29	32	2.2	***	ns	ns

DEB did not affect the nitrogen balance (Table 4), although protein efficiency (PE) was higher in trout fed the DEB 200 diet (P < 0.05). In addition, DO level affected gross nitrogen (P < 0.01, GN) and digestible nitrogen intake (P < 0.01, DN), branchial and urinary nitrogen loss (P < 0.01, BUN) and retained nitrogen (P < 0.05, RN), but did not affect PE. Normoxic fish had higher DN (41%) and RN (42%) than hypoxia groups.

DEB affected heat production (P < 0.01) and metabolizable energy for maintenance (P < 0.001) (Table 5). Heat production and energy requirements for maintenance were 26% and 65% higher, respectively, in DEB 700 diets than in DEB 200 diets. On the other hand, DO levels affected all energy balance parameters (P < 0.01), with the exception of maintenance. Gross energy (GE) and digestible energy intake (DE) were 43% and 40% higher respectively in the normoxic fish. Metabolizable energy (ME), heat production (HP) and retained energy (RE) were also 40%, 23% and 54%, respectively, higher in the normoxic fish. The DO levels affected energy retention in the form of lipids (P < 0.01) and protein (P < 0.05). Normoxic group retained more energy as lipid (64%) and protein (42%) than hypoxic group.

### Changes in blood and chyme parameters

**Figure 2:**
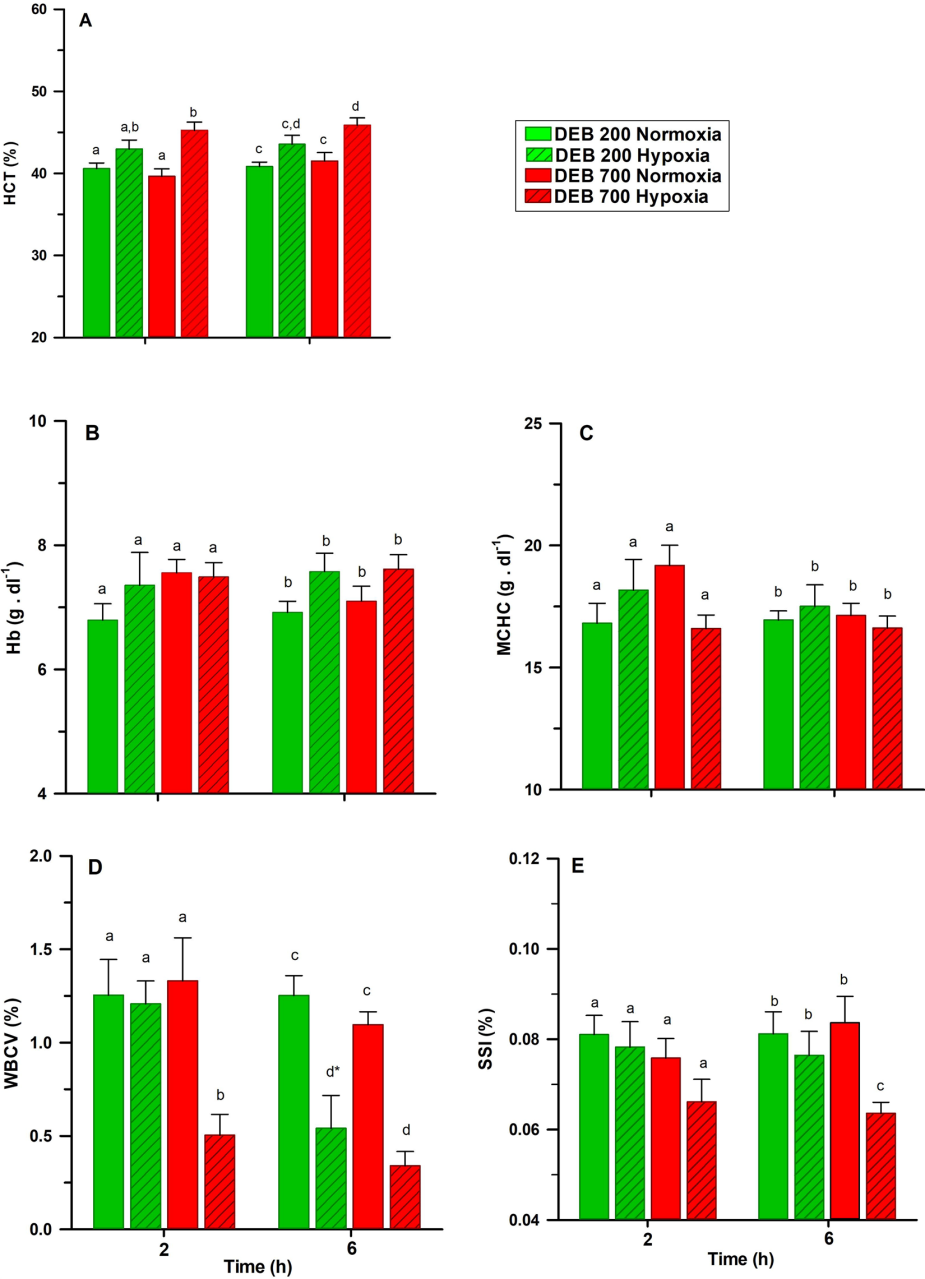
Effects of dietary electrolyte balance (DEB) and dissolved oxygen levels on (**A**) hematocrit (HCT); (**B**) haemoglobin (Hb); (**C**) mean corpuscular haemoglobin concentration (MCHC); (**D**) white-blood cell volume (WBCV) and (**E**) spleno-somatic index (SSI) of rainbow trout 2 or 6 h after feeding. Further details are provided in Methods. Values are mean ± SEM (n = 9). Different letters indicate differences among treatments, while the asterisk indicates differences between sampling times (α = 0.050).

The effects of DEB and DO levels on the O_2_-carrying capacity at 2 and 6-h post-prandial are presented in Fig. 2, and the statistical analyses are presented in Table 6. Dietary treatment has no effect on HCT, Hb or MCHC. An interaction between the diet and DO levels for MCHC was observed (P < 0.05). Hypoxic fish fed the DEB 700 diet had lower SSI and WBCV than fish fed the DEB 200 diet (P < 0.05) 6-h after feeding. HCT were higher (P < 0.001) and WBCV lower (P < 0.001) in the hypoxic fish than in the normoxic fish, regardless of the dietary treatment. The effect on WBCV was more pronounced in fish fed the DEB 700 diet, at 6-h after feeding (P < 0.05), and an interaction between diet and DO levels was observed (P < 0.05).

**Table 6:** Three-way ANOVA analysis on the effects of diet (DEB), dissolved oxygen levels (DO) and time after feeding (T) and their respective interactions on blood parameters and spleen-somatic index. Hematocrit (HCT); Haemoglobin (Hb); Mean corpuscular haemoglobin concentration (MCHC); White-blood cell volume (WBCV); Spleen-somatic index (SSI). Not significant (ns) P > 0.1; *P < 0.05; **P < 0.01; ***P < 0.001.

Parameters	Factors			Interactions			
	DEB	DO	T	DEB-DO	DEB-T	DO-T	DEB-DO-T
HCT	ns	***	ns	ns	ns	ns	ns
Hb	ns	*	ns	ns	ns	ns	ns
MCHC	ns	ns	ns	*	ns	ns	ns
WBCV	*	***	*	*	ns	ns	ns
SSI	*	**	ns	ns	ns	ns	ns

**Figure 3:**
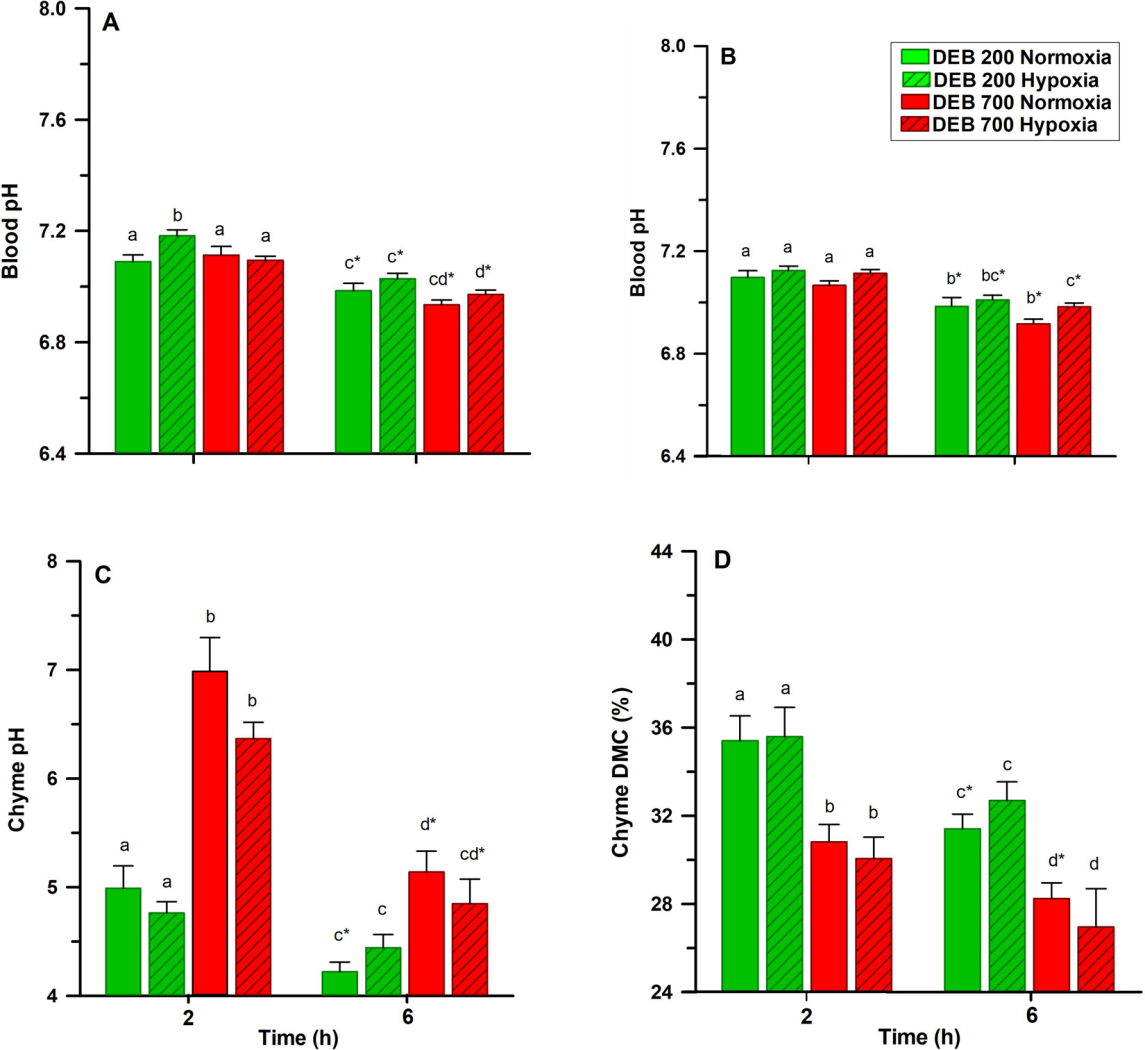
Effects of dietary electrolyte balance (DEB) and dissolved oxygen levels on (**A**) heart blood pH; (**B**) caudal blood pH; (**C**) chyme pH, and (**D**) dry matter content in the stomach of rainbow trout 2 or 6 h after feeding. Further details are provided in Methods. Values are mean ± SEM (n = 9). Different letters indicate differences among treatments, while the asterisk indicates differences in sampling times (α = 0.050).

The post-prandial effects of DEB and DO levels on blood pH and chyme characteristics in trout are presented in Fig. 3 and the statistical analysis in Table 7. Diet had a clear effect on blood pH, both in the heart and the caudal region. The pH was significantly lower in heart (P < 0.01) and the caudal region (P < 0.05) in fish fed the diet with alkaline properties (DEB 700). Conversely, the chyme pH was significantly higher in fish fed the DEB 700 diet (P < 0.001). The chyme dry matter was lower in fish fed DEB 700 (P < 0.001) when compared with DEB 200 (Fig. 3). DO levels had a significant effect on blood pH. Fish subjected to hypoxic conditions had a higher blood pH values when measured in the heart (P < 0.05) and in the caudal region (P < 0.001). However, chyme pH and DM were not affected by DO levels. Stomach chyme pH was significantly affected by the interaction between diet post-prandial time (P < 0.001). Blood pH, stomach chyme DM and pH decreased over time after feeding (P < 0.001).

**Table 7:** Three-way ANOVA analysis on the effects of diet (DEB), dissolved oxygen levels (DO) and time after feeding (T) and their respective interactions on blood and chyme selected parameters. ^1^Obtained from the heart. ^2^Obtained from the caudal region. Not significant (ns) P > 0.1; *P < 0.05; **P < 0.01; ***P < 0.001.

Parameters	Factors			Interactions			
	DEB	DO	T	DEB-DO	DEB-T	DO-T	DEB-DO-T
Blood pH^1^	**	*	***	ns	ns	ns	ns
Blood pH^2^	*	**	***	ns	ns	ns	ns
Chyme pH	***	ns	***	ns	***	ns	ns
Chyme DMC	***	ns	***	ns	ns	ns	ns

## Discussion

The current study investigated the metabolic response of an isogenic rainbow trout line (R23) chronically exposed to a combination of nutritional (electrolyte-imbalanced diet, DEB 700 mEq Kg^−1^) and environmental (hypoxia, 4.5 ± 0.1 mg O_2_ l^−1^) stressors, by analysing changes on FI, growth performance, nutrient digestibility and energy balance. Feeding rainbow trout an electrolyte-balanced diet (DEB 200) caused lower metabolizable energy requirements for maintenance (MEm) when compared with fish fed the electrolyte-imbalanced diet (DEB 700), without significant changes in FI itself, growth performance, or oxygen consumption. The increase of MEm in the DEB 700 group may indicate an increase (65%) in the energy usage to maintain acid-base balance. A comparable increase in MEm values (54%) was observed in Nile tilapia fed the DEB 800 diet, when compared to fish fed the DEB 200 diet^24^. In contrast to the current study, Saravanan *et al*.^24^ detected that feeding a DEB 800 diet to Nile tilapia for 35 days caused a 15% decrease in growth rate.

In spite of an increase in the energy use in trout fed the DEB 700 diet, FI remained similar in both dietary treatments. This was surprising, as higher FI was expected to occur in trout fed the DEB 200 diet. This assumption was based on an expected higher O_2_ availability (larger scope for growth) in trout consuming the DEB 200 diet, as an increased O_2_ demand will be anticipated in fish fed electrolyte-imbalanced diet (DEB 700) to keep the fish’ homeostasis. As previously observed in trout, a decrease in FI with the increase in the dietary energy (lipid) content, may reflect a reduced scope for growth. Thus, factors affecting O_2_ availability may determine the limits in FI^8,26^ (a.k.a. oxystatic concept for FI). Therefore, results from this study do not correspond with the oxystatic concept. Nevertheless, the lack of differences in FI may be related to differences in diet composition, such as a higher palatability due to increased Na_2_CO_3_/Diamol content in the DEB 700 diet. Although no noticeable differences were observed in the feeding behaviour of trout fed DEB 200 or DEB 700 diets, future experiments should further investigate the effects of dietary electrolyte composition on diet palatability.

As expected, normoxic fish displayed higher oxygen consumption than the hypoxic group, indicating limited oxygen availability, which constrains FI in trout. Hypoxia was reflected in a 35% decrease in growth and FI when compared to the normoxic group. Results are consistent with previous reports showing decreased FI due to reduced O_2_ availability in rainbow trout^5,6^. On the other hand, feed conversion ratio (FCR) and efficiency in the utilization of dietary digestible protein (PE) were not affected by DO levels, confirming the previous observations in rainbow trout^5^. However, Glencross *et al*.^5^ have shown larger decreases in growth (54%) and FI (46%) in hypoxic trout (5.7 ± 1.4 mg O_2_ l^−1^) compared to the group in normoxia (9.3 ± 0.4 mg O_2_ l^−1^). Such differences may be due to the use of smaller trout (approximately half the size) by Glencross *et al*. compared to this study, rather than a differential response of the isogenic trout line to low DO levels. It has been suggested that low DO levels could have a stronger impact in bigger fish, posing a more strict limitation on FI and growth due to the allometric relationship between the gill surface area and BW^27^.

Feeding trout the electrolyte-imbalanced diet (DEB 700) did not result in changes in oxygen consumption, as the value for this parameter remained similar between both dietary groups (2.4 and 2.0 mg O_2_ kg^−0.8^ min^−1^ in normoxia and hypoxia, respectively). These results are in line with a previous study by Saravanan *et al*.^13^ in which oxygen consumption was identical in trout fed amino-acid-balance or -imbalance diets under normoxic (2.8 mg O_2_ kg^−0.8^ min^−1^) or hypoxic (2.4 mg O_2_ kg^−0.8^ min^−1^) conditions. Greater oxygen consumption measured by Saravanan *et al*.^13^ for both environmental DO levels may be due to the use of smaller trout than in our study (half BW), as similar conditions in the aquatic metabolic unit were implemented in both studies regarding the contrast in DO between hypoxia and normoxia.

Results showed that trout in normoxia displayed higher values for all the nitrogen (GN, DN, BUN, RN) and energy balance parameters (GE, DE, BUE, ME, RE, HP) than in hypoxia. However, normoxic fish showed lower values for all the digestibility parameters analysed (protein, carbohydrates, lipids and energy) than in the hypoxic group. These results suggested that rainbow trout was able to increase the efficiency of nutrient digestibility when chronically exposed to low DO levels. However, protein utilization in trout remained similar under different DO levels, which suggest an increased reliance on carbohydrates, and lipids to less degree, as energy sources under hypoxic conditions. Similarly, Glencross *et al*.^5^ found that protein utilization remained similar in trout under hypoxia. They suggested that hypoxic trout may increase the energy efficiency by decreasing the energy allocated toward lipid synthesis, as lipid metabolism is more sensitive to oxygen availability as an energy source. We propose that hypoxic trout may be able to partially compensate the decrease in energy availability, as a consequence of reduced FI, by increasing the nutrient digestibility for all macronutrients. Such compensation may also involve an increased efficiency of metabolic pathways related with carbohydrate and lipid metabolism during hypoxia. The proposed increases in both digestibility and metabolic energy efficiencies in fish exposed to chronic hypoxia requires further investigation to distinguish their potential contribution toward achieving energy balance.

Fish liver has a central role supplying energy to the tissues, with a remarkable capacity to store and mobilize energy reserves in response to several environmental and nutritional cues^28^. In particular, the hepatosomatic index (HSI) is a useful tool as a means of appraising metabolic capacity. For example, low levels of the essential amino acid lysine in experimental diets were reflected in higher HSI in rainbow trout^29^. In our study, the HSI of trout increased by 32% in normoxia, and 10% in hypoxia, when fed the alkaline diet. These changes suggest that trout fed the DEB 700 diet displayed a different metabolic capacity at a hepatic level, probably reflecting the increased energy demand necessary to maintain acid-base balance.

Regarding effects of the diet on nutrient digestibility, our study showed that apparent digestibility of DM and ash in trout fed the DEB 700 diet was higher than in the DEB 200 group. This positive effect on nutrient digestibility could be attributed to an increased fluid secretion into the stomach, with higher food liquefaction and lower chyme pH in the intestine of fish fed the alkaline diet, as has been previously hypothesized in the Nile tilapia by Saravanan *et al*.^24^. This interpretation is reinforced by the lower chyme DM content in the stomach of trout 2 and 6 hs after being fed the DEB 700 diet when compared to the DEB 200 group.

Higher Diamol content in the DEB 200 diet may have reflected in larger ash content of the faeces in fish fed this diet compared to the DEB 700 group. If ash digestibility is corrected for Diamol addition, assuming it to be fully inert, the ADC for ash is 42.9 and 54.0% for the DEB 200 and DEB 700 diets, respectively. The difference in ash ADC might furthermore be caused by leaching of Na_2_CO_3_ from the feed prior to consumption of the pellet. However complete leaching of Na_2_CO_3_ was not very likely because fish were consuming at least 75% of the feed very quickly and also due to the presence effects DEB on post prandial blood pH.

Previous studies have shown that there is a very large addition of fluid to the chyme in the stomach of trout, which continues as the digestion proceeds by endogenous water addition and/or secretion of digestive fluids in the gastro-intestinal tract of fish^30–32^. Regarding the mechanisms that may be involved, it has been previously suggested a higher liquefaction of the chyme in the stomach of fish fed high DEB levels can be due to altered osmolality, promoting the hydration of the content with endogenous water by osmosis, and/or increased secretion of digestive fluids^24^. Liquefaction of a dry feed in the stomach may produce a feed consistency resembling the natural feed, which may facilitate the digestive processes, as the chyme DM content is inversely related to nutrient digestibility^32^.

Apparent digestibility for phosphorus, lipid and energy were lower in fish fed the alkaline diet (DEB 700). This is surprising, as a previous study in Nile tilapia showed that increasing DEB levels had a positive effect on the digestibility of nutrients, which could partially compensate the increased maintenance requirements induced by highly alkaline diets^24^. In contrast to our study, Saravanan *et al*. showed that lipid digestibility was unaffected by DEB levels. Differences in the digestibility between both studies may be entirely due to the different feeding method employed, as tilapia were fed restrictively^24^, whereas our trout were fed to apparent satiation. However, the changes detected in trout’s digestibility could be due to a higher DM content of the DEB 700 as well, which may require higher liquefaction, lowering the intestinal chyme pH and decreasing the efficiency of digestive lipases. Also differences in chyme characteristics between both diet groups (e.g. stomach pH; Fig. 3) could be responsible for the observed differences in the phosphorus digestibility.

The isogenic trout line responded to long term low DO levels by boosting their O_2_-carrying capacities, mainly by increasing the hematocrit values. Results are in agreement with previous studies showing an enhancement on the O_2_-carrying capacity of rainbow trout under environmental hypoxia^33,34^. Splenic contraction and subsequent erythrocyte release into the circulation has been shown to occur in fish after acute hypoxia^35^. A previous study in rainbow trout has shown that the spleen remained contracted (lower SSI) when exposed to hypoxia (approximately 3.23 mg O_2_ l^−1^ DO), although SSI values return to normal after 6 days^36^. In our study, the SSI in trout was not significantly different between normoxic and hypoxic conditions when fed the DEB 200. However, lower SSI values were found in hypoxic trout 6 hs after being fed the DEB 700 diet. Decreased SSI values may be explained by a reduction on the haemoglobin’s O_2_-carrying capacity due to altered acid-base balance in fish fed the alkaline diet, which will require in counterpart an enhancement of this capacity under hypoxia by the release of erythrocyte from the spleen under hypoxia.

The results of our study showed that nutritional (DEB diets) and environmental (DO levels) factors produced changes in gastric chyme and blood pHs. As expected, the pH of the stomach chyme was higher in fish fed the alkaline diet (DEB 700), changes that were conspicuous even 6 hs after feeding. Alteration of chyme pH was reflected in a lower blood pH in the heart and the caudal region as well, as the fish digested the alkaline diet. Previous studies have shown that the rise in pH and HCO_3_^−^ in arterial blood of trout after feeding (a.k.a. alkaline tide) is compensated by the excretion of base equivalents to the environment^30,37^. Similar results to our study have been observed in Nile tilapia 7 hs after feeding a DEB 800 diet, showing that blood pH in the heart was lower than in fish fed a DEB 200 diet^24^. This response suggests that the prolonged effects of a DEB diet on the systemic acid-base homeostasis of fish triggers mechanisms to fully compensate for the alteration in acid-base balance^38,39^. Therefore, rainbow trout when fed an alkaline diet appears to respond with a reduced alkaline tide, suggesting that the compensatory mechanisms taking place in the gills and kidney were more effective in this group than in fish fed the DEB 200 diet.

In the trout isogenic line, the pH of the blood was significantly increased in response to chronic hypoxic conditions. This contrasts with the blood acidification described by Claireaux *et al*.^40^ in trout exposed to acute hypoxia, with a subsequent increase in the haemoglobin affinity for O_2_. Internal alkalization of erythrocytes at the onset of acute hypoxia was believed to be responsible for the extracellular acidosis observed by Claireaux *et al*.^40^ in trout. However, blood acidification during hypoxia occurred when fish were fasted at least 24 h before measurements, contrasting with our study in which blood parameters in trout were analysed 2 or 6 h after feeding. Differences may due to the effect of digestion on blood pH combined with exposure to chronic hypoxia, as different adaptive mechanisms of the erythrocytes may take place when fasted trout is exposed to acute hypoxia. An interaction between DEB and DO levels was expected to occur in the FI and growth of trout, mainly due to the reduced O_2_-carrying capacity of haemoglobin in the DEB 700 group, but these effects were not observed in this study. This may be explained by the presence of compensatory mechanisms to the O_2_-binding properties of haemoglobin in response to stressors in rainbow trout^41^. Various forms of stressors have been shown to reduce the numbers and functions of white blood cells in fish^42^. We found decreases in the white blood-cell volume (WBCV) of trout exposed to hypoxia or when fed the DEB 700 diet. These observed decreases in WBCV were probably linked to higher hematocrit values detected in trout subjected to these treatments, although the mechanisms mediating both responses remain to be investigated.

In conclusion, FI was not affected by DEB in rainbow trout, but was decreased by long-term hypoxic conditions. DEB affected acid-base balance, as an electrolyte-imbalanced diet (DEB 700) required an increased energy expenditure to maintain acid-base homeostasis, although oxygen consumption remained unaffected in trout. Stomach chyme pH was higher in trout fed the alkaline diet (DEB 700), reflecting in lower blood pH in the heart and the caudal region as the digestion proceeded. Fish fed the DEB 700 responded with a reduced alkaline tide, suggesting that compensatory mechanisms were effective in reducing the impact triggered by the acid-base imbalance. As a result, feeding trout an electrolyte imbalanced diet resulted in significantly higher requirements for metabolizable energy for maintenance (MEm) than a diet with a better electrolyte balance (DEB 200). However, increased energy expenditure used towards acid-base regulation caused by the DEB 700 diet did not alter growth. This could be due to slight differences in FI, undetected in the present study, which may have compensated for a higher MEm of the DEB 700 diet.

This study shows the importance that dietary electrolyte balance (e.g. mineral content) and DO levels have on energy balance and growth performance when fish are fed to satiation. Understanding the factors affecting FI may assist to improve management and practical conditions of fish farming, for example by adjusting feed composition or rearing conditions. This is highly relevant as fish oxygen carrying capacity under intensive aquaculture systems may be changed by the use of novel feed ingredients and different rearing conditions, which ultimately impact FI.

## Methods

### Fish and housing

An isogenic heterozygous family of rainbow trout (R23), produced by crossing two homozygote isogenetic lines (GABI/La Peima, INRA, France)^43^, was used in this study. Fish were housed in the Aquatic Metabolic Unit (AMU) tanks of Aquaculture and Fisheries group, Wageningen University, The Netherlands. The tanks were connected to a common water recirculation system consisting of a trickling filter, an oxygenation unit, a sump, a drum filter (Hydrotech 500®) and a cooling/heating system for maintaining uniform water quality throughout the study. The oxygenation unit maintained the DO levels by injecting oxygen into the water, and was facilitated with separate automatic probes for the detection of water flow and oxygen consumption and was also equipped with faecal collectors for measuring digestibility as described by Saravanan *et al*.^14^. Water temperature was set at 14 ± 1 °C. Photoperiod was maintained at 12:12 (Light: Dark) with daybreak set at 07:00 h.

### Experimental design

Rainbow trout were housed in the AMU, according to a 2 × 2 factorial design, with diet (DEB 200 or DEB 700) and water DO levels (normoxia or hypoxia) as factors. Twelve experimental tanks (200 L each) were divided into three blocks of four tanks in each block, and the four treatments were assigned randomly within each of three blocks (N = 3 tanks treatment^−1^). Three hundred and seventy fish were weighed (115.2 ± 2.0 g) and 30 fish were randomly assigned to each of the 12 tanks. Ten fish were randomly sampled for initial body composition analyses.

### Experimental diets and feeding

Two isoproteic (45% DM) and isoenergetic (22 kJ gDM^−1^) diets (Table 8) were extruded by Research Diet Services (Wijk bij Duurstede, The Netherlands). The diet was a floating 4 mm pellet and contained 0.01% yttrium oxide as inert marker for the determination of the apparent digestibility coefficient. The two diets were formulated to provide a contrast in electrolyte content (DEB); 200 or 700 mEq Kg^−1^. This difference was created by adding different amounts of Na_2_CO_3_ and diamol (inert filler) in the diets.

**Table 8:** Ingredients and proximate composition of the experimental diets. DEB, dietary electrolyte balance (mEq kg^−1^). *Diamol GM; Franz Bertram. ^¶^RE > 680.

Test ingredients (%)	DEB 200	DEB 700
Na_2_CO_3_	0.3	2.9
Diamol*	2.7	0.1
Wheat	27.2	27.2
Wheat gluten	13.0	13.0
Fish meal^¶^	13.0	13.0
Fish oil^&^	14.0	14.0
Soya protein concentrate	13.0	13.0
Pea protein concentrate	13.0	13.0
Lysine HCL	0.3	0.3
DL-methionine	0.5	0.5
Monocalcium phosphate	1.5	1.5
CaCO_3_ (krijt)	0.5	0.5
Yttrium oxide	0.01	0.01
Premix^£^	1.0	1.0
Total	100.0	100.0
**Proximate composition**		
DM (%)	93.0 ± 0.1	92.6 ± 0.2
Ash (% on DM)	11.2 ± 0.1	10.6 ± 1.2
Crude protein (% on DM)	45.0 ± 0.1	45.2 ± 0.3
Crude lipid (% on DM)	15.3 ± 0.2	16.0 ± 0.2
TC (% on DM)	31.6 ± 0.2	31.5 ± 0.1
GE (kJ g DM^−1^)	22.0 ± 0.1	22.4 ± 0.2

^£^Vitamin premix composition (to supply, mg/kg feed): 10, B1; 10, B2; 20, B3; 40, B5; 10, B6; 0.2, biotin; 2, folic acid; 0.015, B12; 2000, choline (as choline chloride); 100, C (as ascorbic acid C phosphate); 3000 IU, A (as A palmitate), 2400 IU, cholecalciferol (Rovimixw D3-500; DSM, Inc.); 100 IU, E; 10, menadione (as menadione sodium bisulfite, 51%); 400, inositol; 100, antioxidant BHT (E300-321); 1000, calcium propionate. Mineral premix composition (to supply, mg/kg feed): 50, Fe (as FeSO_4_.7H_2_O); 30, Zn (as ZnSO_4_.7H_2_O); 0.1, Co (as CoSO_4_.7H_2_O); 10, Cu (as CuSO_4_.5H_2_O); 0.5, Se (as Na2SeO3); 20, Mn (as MnSO_4_.4H_2_O); 500, Mg (as MgSO_4_.7H_2_O); 1, Cr (as CrCl_3_.6H_2_O); 2, I (as CaIO_3_.6H_2_O). DM, dry matter; TC, total carbohydrates; GE, gross energy. Proximate composition values are presented as mean ± SEM (n = 3). No statistical differences were found for any of parameters analysed between the diets (P > 0.05).

Fish were fed the experimental diets to apparent satiation, twice a day for 42 days. At the end of each feeding session, feed given and uneaten feed pellets were counted to determine FI on a daily basis. Faeces were collected to determine nutrient digestibility in a similar manner as described by Amirkolaie *et al*.^32^.

### Experimental conditions

Experimental tanks, connected to a common water recirculation system, had an oxygenation unit supplying water at a constant DO level (10.2 ± 0.1 mg O_2_ l^−1^) by injecting pure oxygen into the common inlet, which was regulated by a mass flow controller (Brooksw Model 5850 S; Brooks Instruments) and a microprocessor (Brooksw Read Out and Control Electronics Model 0154; Brooks Instruments). Each metabolic tank was equipped with a water flow meter (MAGFLOWw MAG 5000; Danfoss A/S) to regulate and monitor water flow (inlet). The volume of water within the tanks was kept identical (200 litres) by adjusting a standpipe. The water surface of each tank was covered with a water-resistant floating panel to prevent gas exchange between water and air. Within the floating panel, a circular feeding hatch (18·5 cm in diameter) with a removable floating lid was used to feed the fish.

The outlet of each metabolic tank was linked to a measuring hub to continuously measure DO (WTW-Trioximaticw 700 IQ; WTW GmbH), pH (WTW-SensoLyt DWw (SEA) 700 IQ; WTW GmbH) and conductivity (WTW TetraCon325w 700 IQ; WTW GmbH) of water. Measured values of DO, pH and conductivity were automatically recorded in a personal computer.

The difference in DO levels was induced by adjusting the rate of water inflow to the tanks as described by Saravanan *et al*.^13^. On this procedure, in the normoxic groups, the rate of water inflow to each tank was kept at 7.2 ± 0.0 l min^−1^ (mean ± SEM) with a mean water DO level of 10.2 ± 0.1 mg O_2_ l^−1^. The DO level in the outflowing water remained at 7.9 ± 0.1 mg O_2_ l^−1^. Hypoxia conditions were created by gradually reducing the rate of water inflow to the tank to reach a water flow of 2.2 ± 0.0 l min^−1^ of oxygenated water (10.2 ± 0.1 mg O_2_ l^−1^) for the first three days after the start of the experiment. After that time, the DO level in the outflowing water for each hypoxic tank was maintained at 4.5 ± 0.1 mg O_2_ l^−1^ until the end of the trial.

Critical oxygen tension for rainbow trout (P_crit_) has been reported to be 2.9 kPa at 15 °C^44^, 3 times lower than the O_2_ level used in this study (4.5 ± 0.1 mg O_2_ l^−1^ or 9.1 kPa at 14 °C). However, the DO level applied in the hypoxia treatment is recognized as an environmental challenge, with the value decided based on the reported critical DO level of 6.0 mg O_2_ l^−1^ for feed consumption and 7 mg O_2_ l^−1^ for both growth rate and feed conversion efficiency rainbow trout at 15 °C^6^.

The oxygen consumption of fish was monitored throughout the entire experimental period. Oxygen content in the inlet and outlet of each tank was automatically measured at 5 min intervals using an electrode (WTW-TrioximaticH 700 IQ, WTW GmbH, Weilheim, Germany) and data was recorded in a personal computer using an interface (HTBasic, Version 9.5, TransEra Corp.). The oxygen electrode was calibrated once a week.

### Fish sampling

Fish were sampled at the start and at the end of the trial to determine initial (n = 10) and final body composition (n = 10 per tank), respectively. At 42 days of trial, fish were sampled at 2 h and 6 h after the morning feeding. Prior to sampling, fish were anesthetized with an overdose of 2-phenoxy ethanol (ml l^−1^) and then blood was drawn from the heart and caudal blood vessels with a heparinized syringe. Blood pH was immediately measured (pH meter, WTW pH 320; pH electrode, WTW SenTix Sp). The duration of anaesthesia (2 min) and the fish-handling period were strictly standardized for all fish to minimize blood pH variation^24^. After pH measurement, blood samples were used to measure hematocrit (HCT), white blood cell volume (WBCV) values, and haemoglobin (Hb) concentration. Fish were weighed and euthanized to sample gastric chyme, liver and spleen. Samples were frozen in liquid nitrogen and stored at −80 °C for later analyses.

### Chemical Analysis

Whole fish from each tank (3 fish tank^−1^) were ground, pooled and fresh moisture content was determined. Fish and faeces were subsequently freeze-dried before further analyses. Feed, faeces and whole-body samples were analysed in triplicates for dry matter (105 °C for 24 h) and protein (Kjeldahl; N ∙ 6.25) after acid digestion. Lipid content of feed and faeces were analysed as described by Folch *et al*.^45^ and by petroleum ether extraction (Soxhlet; 40–60 °C) in the whole fish. Gross energy content was analysed in an adiabatic bomb calorimeter (IKA-Werke C5000). Ash content was determined by combustion in muffle furnace (550 °C for 12 h). The same ash samples of feed and faeces were used to determine acid insoluble ash^46^. Yttrium content in feed and faeces was measured by inductively coupled plasma atomic emission spectroscopy (ICP-AES)^47^.

### Measurements and calculations

The percentage of fish survival (S) was calculated as:

1 $${\rm{S}}( \% )=[{{\rm{N}}}_{{\rm{f}}}/{{\rm{N}}}_{{\rm{i}}}]\times 100$$

where N_f_ is the final number of fish and N_i_ is the initial number of fish.

Mean metabolic weight of fish (MBW_g_) was calculated as:

2 $${{\rm{MBW}}}_{{\rm{g}}}({{\rm{kg}}}^{0.8})={[{{\rm{W}}}_{{\rm{g}}}/1000]}^{0.8}$$

where W_g_ is the geometric mean body weight (g).

FI per unit of mean metabolic body weight (FI_MBW_) was calculated as:

3 $${{\rm{FI}}}_{{\rm{MBW}}}({{\rm{gDMkg}}}^{-0.8}{{\rm{day}}}^{-1})={{\rm{FI}}}_{{\rm{ABS}}}/{{\rm{MBW}}}_{{\rm{g}}}$$

where FI_ABS_ is the daily absolute FI, calculated as:

4 $${{\rm{FI}}}_{{\rm{ABS}}}({\rm{gDM}}\,{{\rm{fish}}}^{-1}\,{{\rm{day}}}^{-1})={{\rm{FI}}}_{{\rm{TOT}}}/{\rm{n}}\times {\rm{t}}$$

where FI_TOT_ is the total FI per tank (g DM) over the experimental period corrected for dead fish and uneaten feed, n is the number of fish per tank and t is the experimental period (days).

Growth rate on metabolic weight (GR_MBW_) was calculated as:

5 $${{\rm{GR}}}_{{\rm{MBW}}}({\rm{g}}\,{{\rm{kg}}}^{-0.8}\,{{\rm{day}}}^{-1})=[{{\rm{W}}}_{{\rm{f}}}-{{\rm{W}}}_{{\rm{i}}}]/[{{\rm{MBW}}}_{{\rm{g}}}\times {\rm{t}}]$$

where W_f_ is the mean final BW (g), W_i_ is the mean initial BW (g) and t is the duration of the growth study (days).

Feed conversion ratio (FCR) was calculated as:

6 $${\rm{FCR}}={\rm{FI}}({\rm{g}}\,{\rm{DM}})/[{{\rm{W}}}_{{\rm{f}}}-{{\rm{W}}}_{{\rm{i}}}]({\rm{g}}\,{\rm{wet}}\,{\rm{weight}}\,{\rm{gain}})$$

The hepato-somatic index was calculated as:

7 $${\rm{HSI}}\,( \% )=100\times [{\rm{liver}}\,{\rm{weight}}\,({\rm{g}})/{\rm{BW}}({\rm{g}})]$$

The spleen-somatic-index was calculated as:

8 $${\rm{SSI}}\,( \% )=100\times [{\rm{spleen}}\,{\rm{weight}}\,({\rm{g}})/{\rm{BW}}\,({\rm{g}})]$$

Mean corpuscular haemoglobin concentration was calculated as follows:

9 $${\rm{MCHC}}({\rm{g}}\,{{\rm{dl}}}^{-1})=([{\rm{Hb}}]/{\rm{Hct}})\times 100$$

where [Hb] is the concentration of haemoglobin in blood (g dl^−1^) and Hct the hematocrit value (%).

Total carbohydrate content in diets and faeces were calculated as:

10 $${\rm{Total}}\,{\rm{carbohydrate}}\,({\rm{DM}} \% )=100-[{\rm{Crude}}\,{\rm{protein}}+{\rm{Crude}}\,{\rm{lipid}}+{\rm{Ash}}]$$

The apparent digestibility coefficients (ADC) of protein, lipid, total carbohydrates and energy were calculated by comparing the amount of yttrium (Y) as an inert marker in relation to the content of the nutrient in the feed and faeces, according to:

11 $${{\rm{ADC}}}_{{\rm{X}}}({\rm{DM}} \% )=[1-({{\rm{Y}}}_{{\rm{diet}}}/{{\rm{Y}}}_{{\rm{faeces}}})\times ({{\rm{X}}}_{{\rm{faeces}}}/{{\rm{X}}}_{{\rm{diet}}})]\times 100$$

where X represents dry matter, crude protein, crude lipid, total carbohydrate, gross energy or ash, Y_diet_ and Y_faeces_ are the yttrium content in the diet and faeces, respectively, and X_diet_ and X_faeces_ are the X content in diet and faeces, respectively.

The gross nitrogen intake (G_N_) was calculated as:

12 $${{\rm{G}}}_{{\rm{N}}}({\rm{mg}}\,{\rm{N}}\,{{\rm{fish}}}^{-1}\,{\rm{day}})={{\rm{FI}}}_{{\rm{tot}}}({\rm{g}}\,{\rm{DM}}\,{{\rm{fish}}}^{-1}\,{\rm{day}})\times {\rm{N}}\,{\rm{in}}\,{\rm{feed}}\,({\rm{mg}}\,{\rm{N}}\,{{\rm{g}}}^{-1})$$

The digestible nitrogen intake (D_N_) was calculated as:

13 $${{\rm{D}}}_{{\rm{N}}}({\rm{mg}}\,{\rm{N}}\,{{\rm{fish}}}^{-1}\,{\rm{day}})={{\rm{G}}}_{{\rm{N}}}\times {{\rm{ADC}}}_{{\rm{N}}}( \% )$$

The retained N (R_N_, mg N fish^−1^ day) was calculated as the difference between the N content of the final fish carcass and that of the initial fish carcass. Branchial and urinary N loss (BU_N_) was calculated as the difference between D_N_ and R_N_.

Protein efficiency (PE) was calculated as:

14 $${\rm{PE}}\,( \% )=[{R}_{N}\times 6.25]/[{{\rm{G}}}_{{\rm{N}}}\times 6.25]\times 100$$

Oxygen consumption of the fish was calculated per tank with the difference in measured concentration of oxygen between inlet and outlet, and the rate of water flow in the tank using the formula specified by Saravanan *et al*.^14^.

The parameters of energy balance (kJ fish^−1^ day) were calculated as follows: gross energy intake (G_E_) as the product of FI (g DM fish^−1^ day) and energy content of the diet; digestible energy intake (D_E_) as the product of G_E_ and ADC_E_; metabolizable energy intake (M_E_) as the difference between D_E_ and branchial and urinary energy loss (BU_E_), which was estimated as (BU_N_ × 24.85)/1000, where 24.85 is the amount of energy (kJ) equivalent to 1 g excreted N, assuming that all N is excreted as NH_3_-N; retained energy (R_E_) as the difference between the energy content of the final fish carcass and that of the initial fish carcass. Heat production (HP, kJ kg^−0.8^ day) was calculated as the difference between M_E_ and R_E_. Metabolizable energy for maintenance (M_Em_) was estimated as M_E_-(R_E_/0.65), where 0.65 is the efficiency of energy utilization for growth^48^. M_Em_ was divided by W_g_ (kJ kg^−0.8^ day).

### Use of experimental animals

The fish trials were approved and carried out according to the Wageningen University Ethics Board for experimentation with animals (DEC, Registration protocol 2014056.a), under Dutch and EU legislation on the handling of experimental animals.

### Data availability statement format guidelines

The datasets generated during and/or analysed during the current study are available from the corresponding author on reasonable request.
